# Effect of Electropulsing Treatment on Microstructure and Mechanical Properties of a Deformed ZrTiAlV Alloy

**DOI:** 10.3390/ma12213560

**Published:** 2019-10-30

**Authors:** Zhi Nan Yang, Feng Jiang, Xu Biao Wang, Lin Qu, Yan Guo Li, Lin Jiang Chai, Fu Cheng Zhang

**Affiliations:** 1 National Engineering Research Center for Equipment and Technology of Cold Strip Rolling, Yanshan University, Qinhuangdao 066004, China; fengjiang624@126.com; 2 State Key Laboratory of Metastable Materials Science and Technology, Yanshan University, Qinhuangdao 066004, China; wxb19921202@163.com (X.B.W.); lyg@ysu.edu.cn (Y.G.L.); 3 CITIC Dicastal Co., Ltd., Qinhuangdao 066004, China; qulin@dicastal.com; 4 College of Materials Science and Engineering, Chongqing University of Technology, Chongqing 400054, China; chailinjiang@cqut.edu.cn

**Keywords:** metals and alloys, microstructure, phase transformation, mechanical properties, electropulsing treatment

## Abstract

In contrast to conventional heat treatment processes, electropulsing not only heats an alloy, but also exerts some other positive effects during the heating process. In this paper, the microstructural evolution and mechanical properties of a deformed Zr40Ti5Al4V alloy after electropulsing treatment were investigated. The results showed that when the charging voltage was 2 kV, there was a slight decrease in dislocation density due to the electron wind which softened the alloy even though the highest temperature of the specimen during the treatment was only 86 °C. Increasing the charging voltage to 6 kV not only further increased the heating temperature, but accelerated the phase transformation process of α″ → β → α. The presence of the α phase strengthened the alloy but notably deteriorated its ductility. A full and refined β phase microstructure could be obtained when the charging voltage was increased to 8 kV. This simultaneously increased the strength and ductility of the alloy.

## 1. Introduction

Since the 1950s, when the first decision to use zirconium for nuclear applications was made by Admiral Rickover of the US Navy [1], zirconium and its alloys have undergone development [2,3,4]. A series of zirconium alloys with different properties have been developed to satisfy different requirements by changing the alloying elements (e.g., Sn, Nb, Mo, Fe, Al, V, etc.). These elements mainly have a solid-solution strengthening effect on the alloys [5,6,7,8]. Optimizing the heat treatment process is also one effective and conventional method for regulating the properties of alloys. 

The ZrTiAlV alloy is a new zirconium alloy system that can reach a high strength of ~1500 MPa via a certain treatment process [9,10]. The mechanical properties and the microstructural evolution during room-temperature deformation and following the subsequent conventional heat treatment process have been studied [11]. The cooling method also notably influences the microstructure and properties of an alloy [11,12]. The conventional heating process usually needs a long time to reach an equilibrium or para-equilibrium state before the cooling process. In recent years, fast heat treatment processes have gradually become popular [13,14], with the aim of reaching an un-equilibrium state, obtaining a mixed microstructure and improving the alloy’s properties. 

The electropulsing treatment process is an effective fast-heating method, and its positive effect on microstructure and mechanical properties has attracted attention from researchers in the past few decades [15,16,17,18,19,20,21,22,23]. A lot of studies have revealed that the electropulsing treatment process can not only be used to improve the plastic in cold-drawing technology (electroplastic) [15,16], refine the solidified metal microstructure [17,18], improve the fatigue property [19], and arrest cracks [20], it can also refine the microstructure in solid state by improving the velocity of recrystallization and inhibiting the growth of recrystallized grain [21,22,23]. Therefore, the electropulsing treatment process can be introduced in many steps of the manufacturing process of zirconium structural parts in order to improve their properties.

During the conventional heat treatment process of deformed ZrTiAlV alloy, microstructure evolution and phase transformation occur, including recovery and recrystallization, phase transformation from α″ phase to β phase, and, thereafter, from β phase to α phase [11]. In contrast, it is not known how microstructure and mechanical properties would evolve if a fast heat treatment process was used instead of conventional heat treatment. In this paper, the cold rolled β-Zr40Ti5Al4V alloy was treated by high-current-density electropulsing, with different charging voltages. The phase constitution, the microstructure, and the mechanical properties of the treated specimen were characterized for the purpose of investigating the effect of the electropulsing treatment on the microstructure and mechanical properties of the alloy.

## 2. Experimental Procedure

The experimental material, with a composition of ~40.2 wt % Ti, ~4.5 wt % Al, 4.2 wt % V, and balance Zr, was melted in a vacuum consumable-electrode arc furnace and forged into a round bar with a size of Φ 50 mm. The onset and finish transition temperatures from α to β were 550 °C and 670 °C, respectively, for this alloy. Before the deformation process, the forged alloy underwent a solution treatment (ST) in the body-centered cubic (BCC) structure β phase at 850 °C for 1 h, followed by water quenching to produce a single-phase β structure. Then, the alloy was cold-rolled at room temperature to a reduction of 40%. The deformed alloy was machined to plate specimens with a size of 63 mm × 15 mm × 2.86 mm. The specimens were thereafter treated by high-current-density electropulsing with charging voltages of 2 kV, 4 kV, 6 kV, and 8 kV and a pulse duration of 400 μs. The corresponding peak current densities were 0.48, 0.93, 1.44, and 1.92 kA/mm^2^, respectively. Each specimen was treated once.

Microstructural observations were carried out using optical microscopy (OM, Axiover 200MAT, Zeiss, Jena, Germany) and transmission electron microscopy (TEM, JEM-2010, JEOL, Tokyo, Japan). The specimens for OM were first polished and then etched using a mixture of 10 vol % hydrofluoric acid, 5 vol % nitric acid, and 85 vol % distilled water. The specimens for TEM observation were thinned down to a thickness of approximately 40 μm using SiC papers. Final thinning was achieved by electropolishing in an electrolyte mixture containing 10 vol % perchloric acid and 90 vol % methanol. The voltage was kept constant at 13 V, and the polishing was performed at −35 °C. The phase constitution was examined via X-ray diffraction (XRD). The XRD profile of each polished specimen was recorded by a Rigaku D/max-2500 diffractometer (Rigaku, Tokyo, Japan) using Cu Kα radiation. The diffraction profiles were obtained by varying 2θ from 20° to 80° with a step scan of 0.02°. The time spent collecting the data per step was 2 s. Since there were only two detected phases in the treated alloy, the equation of f*_β_* can be expressed as:

$$f_{\beta }=\frac{(1/n)\sum_{j-1}^{n}\left(I_{\beta }^{j}/R_{\beta }^{j}\right)}{(1/n)\sum_{j-1}^{n}\left(I_{\beta }^{j}/R_{\beta }^{j}\right)+(1/n)\sum_{j-1}^{n}\left(I_{\alpha }^{j}/R_{\alpha }^{j}\right)}$$

where *n*, *I*, and *R* represent the number of peaks of the phase used in the calculation, the integrated intensity for a diffraction peak, and the material scattering factor, respectively. *R* can been given by the equation $R=\left(\frac{1}{v^{2}}\right)\left[{\left|F\right|}^{2}p\left(\left(1+\cos^{2}2\theta \right)/\sin\theta \sin2\theta \right)\right]\cdot e^{-2M}$, where *v* is the volume of the unit cell, *F* is the structure factor, and e^−2M^ is the temperature factor, which has a negligible effect on the calculation result. 

Vickers hardness measurements were performed on an FM-ARS 9000 Hardness Tester (Future - Tech, Kawasaki, Japan) after the specimens were polished. A load of 200 g was applied for 10 s at each location. It should be noted that the hardness was measured on the plane of rolling direction (RD)–normal direction (ND). The distance between the neighboring imprints should not be less than three times larger than the size of the imprints. Dog-bone flat tensile specimens with a nominal gauge section of 15 mm × 3 mm × 2 mm (L × W × H) were cut from the rolled plates following electropulsing treatment in RD. Tensile tests were conducted at room temperature using an MTS servo-hydraulic mechanical testing system (MTS, Landmark 100 kN with 370.10 Load Frame) at a strain rate of 5 × 10^−4^ s^−1^, and five tensile tests were carried out for each condition. Fracture surface morphologies of the tensile specimens were examined using scanning electronic microscopy (SEM, Hitachi-4800, Hitachi, Tokyo, Japan ) to characterize the crack propagation mode.

## 3. Results and Discussions

**Figure 1:**
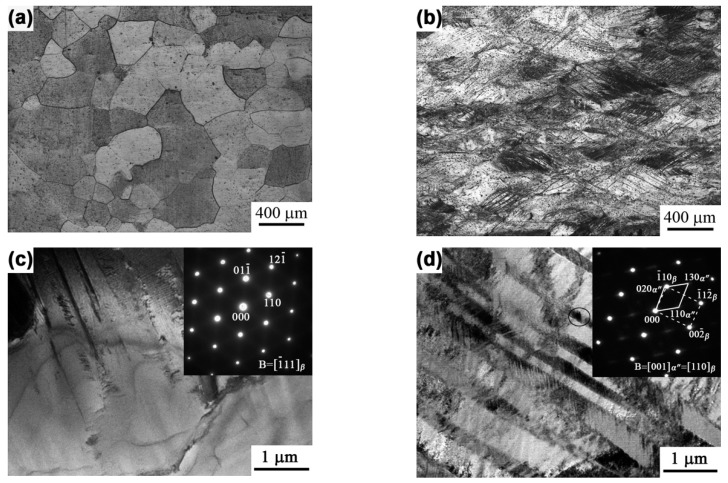
OM and TEM observation of the original specimen (**a**,**c**) and of the 40% rolled specimen (**b**,**d**).

Figure 1 shows the optical and TEM microstructures of the ST and cold-rolled specimens. It can be seen that, after the solutionizing treatment at 850 °C, the alloy presented an equiaxed and full β phase microstructure with a mean grain size of ~346 ± 16 µm. A reduction of 40% caused numerous deformation bands in the stretched grains, as shown in Figure 1b. A detailed characterization of the microstructure via TEM also showed a full β phase microstructure for the original alloy, which was proved by the diffraction pattern shown in Figure 1c. A small number of dislocations that formed during the quenching process were also observed within the β grains. For the cold-rolled specimen, several deformation bands could be seen clearly, with a high density of interior dislocations. Moreover, a large number of thin plates were observed in some bands, which were demonstrated to be α″ phase on the basis of their diffraction patterns, as shown in Figure 1c. A parallel orientation relationship, [001]_α″_ ∕∕ [110]_β_, between the latter α″ phase and the former β phase could also be determined. During the cold-rolling process, the accumulated strain energy provided the driving force for the transformation from the BCC β phase to the orthorhombic α″ phase, which is regarded as displacive transformation [24]. A higher amount of β phase stabilizing the alloying element in the alloy increased the shuffle resistance of the atoms and decreased the atomic shuffle, which resulted in the formation of the transitional phase α″ [11].

**Figure 2:**
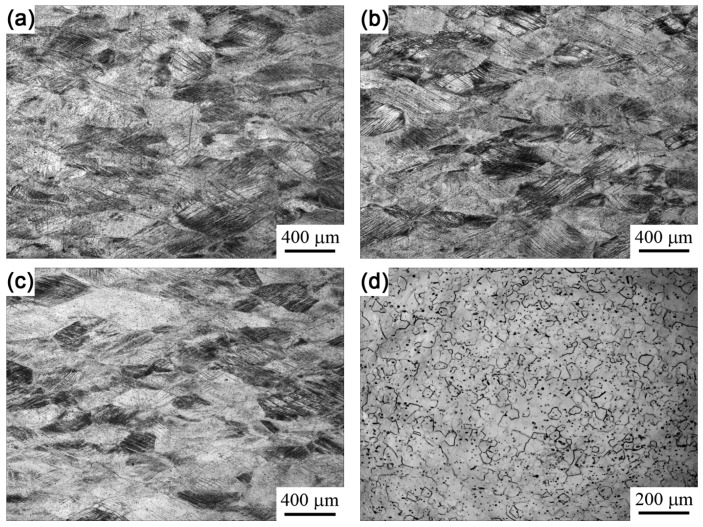
OM microstructure of the specimens after electropulsing treatment at 2 kV (**a**), 4 kV (**b**), 6 kV (**c**), and 8 kV s (**d**).

Figure 2 shows the optical microstructure of specimens treated at various charging voltages. No obvious change could be seen via optical microscopy when the charging voltage was below 8 kV, except for the gradually weakened deformation bands visible when the charging voltage was increased. When the charging voltage was increased to 8 kV, a fully recrystallized microstructure was observed, with a mean grain size of ~26 ± 5 μm, as shown in Figure 2d. The grain appeared notably refined in comparison to the original specimen, as shown in Figure 1a. The grain size of the specimen was also smaller than that obtained via conventional heat treatment, which was ~61 µm [11]. A higher degree of superheat was formed during the electropulsing treatment process, which produced more nucleation sites for recrystallization. Moreover, the extremely short holding time and fast cooling rate after heating inhibited the growth of the recrystallized grain leading to the formation of a refined grain. 

**Figure 3:**
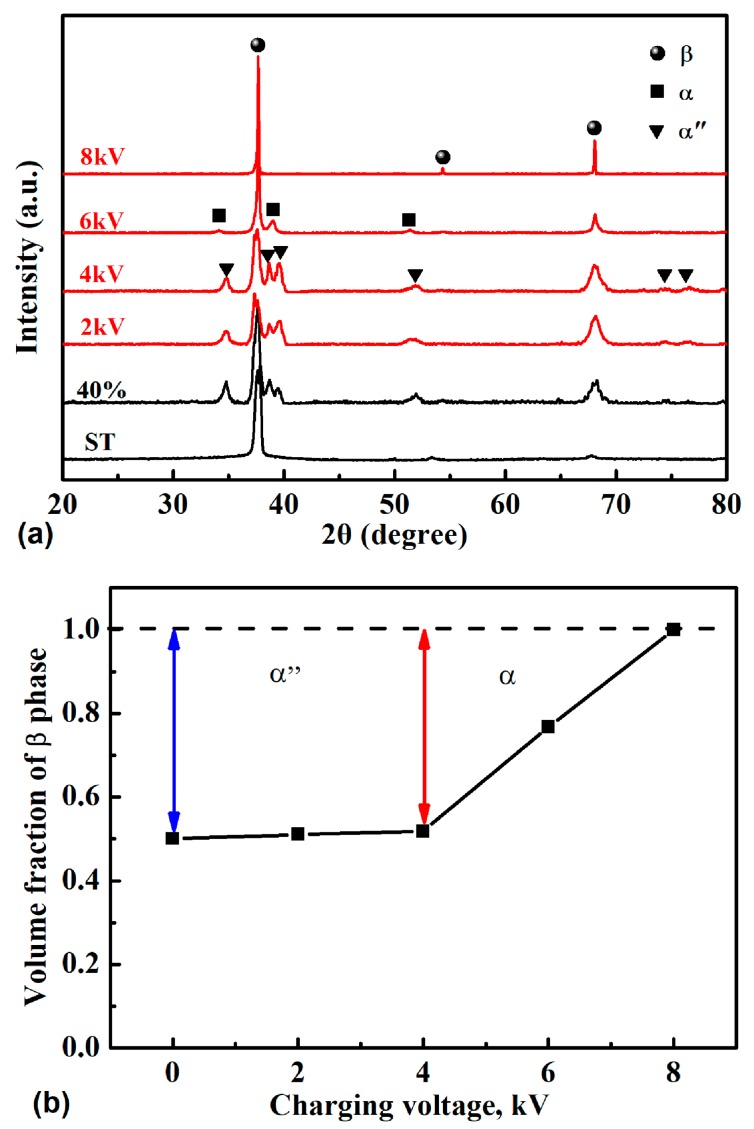
XRD patterns of the solution-treated (ST) specimen, cold-rolled specimen, and electropulsing-treated specimens (**a**) and variation of the volume fraction of the β phase at increasing charging voltage (**b**).

XRD results shown in Figure 3a revealed that the phase constitution in the 2 kV-treated specimen and the 4 kV-treated specimen was nearly the same, with α″ phase and β phase in the microstructure. When the charging voltage was increased to 6 kV, the α″ phase disappeared, and a small amount of α phase formed. With a further increase in the charging voltage to 8 kV, a full β phase was detected. The variation of volume fraction of the β phase is shown in Figure 3b. The evolution of dislocation density in the β phase could be evaluated by detecting the width change of the diffraction peaks of the β phase. There was a clear trend in peak broadening as a function of voltage, as shown in Figure 3a, indicating an increased dislocation density with decreasing charging voltage. The width change was negligible when the charging voltage was less than 6 kV. This change became obvious after a further increase in the charging voltage.

**Figure 4:**
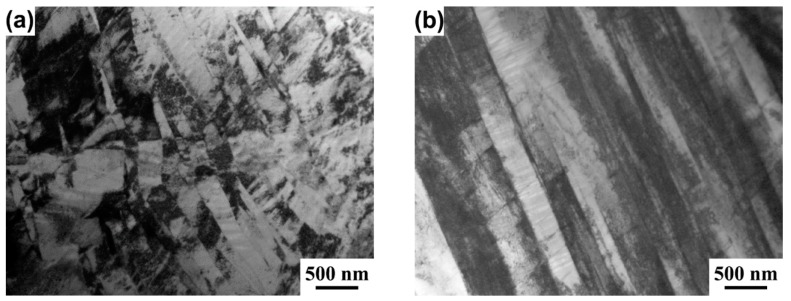

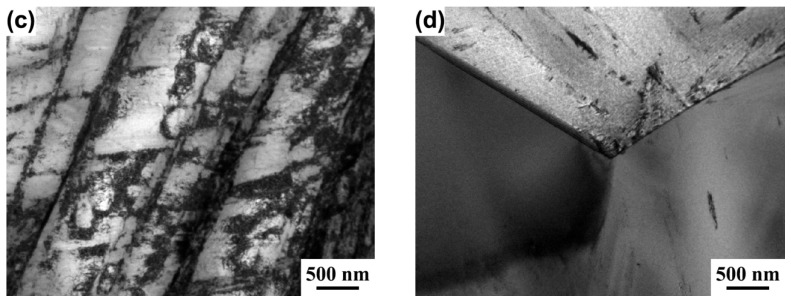
Cross-sectional TEM images of the specimens treated by different charging voltages: 2 kV (**a**), 4 kV (**b**), 6 kV (**c**), and 8 kV (**d**).

TEM observation, as shown in Figure 4, further demonstrated the XRD result. It can be seen that the α″ phase still existed in the microstructure of 2 kV- and 4 kV-treated specimens (Figure 4a,b). Moreover, a high dislocation density was seen in the β plate. With an increase in the charging voltage to 6 kV, a lot of cell sub-structures could be seen in the microstructure, indicating static recovery took place during the short treatment process. A full β phase microstructure was observed after a further increase in the charging voltage to 8 kV. The dislocation density in the β grain was also low, as shown in Figure 4d, in agreement with the XRD result shown in Figure 3a. 

From the above microstructure observations, one can see that the change in microstructure was negligible when the charging voltage was no higher than 4 kV, while it became more obvious in specimens treated at higher voltage. It is well known that the first phase of the electropulsing process results in a fast-increasing temperature. The temperature increment due to the electropulsing process can be calculated with the following formula [25]:

$$\Delta T=\rho {\left(C_{P}dS^{2}\right)}^{-1}\int_{0}^{t_{P}}I^{2}dt$$

where *ρ*, *C_P_*, and *d* are resistivity, specific heat capacity, and density of the alloy, respectively. These physical parameters of the deformed alloy were measured experimentally, resulting in *ρ* = 1.78 × 10^−6^ Ω·m, *C_P_* = 478 J·kg^−1^·°C^−1^, and *d* = 5.20 × 10^3^ kg·m^−3^; *t_p_* is the pulse duration, corresponding to400 μs, *I* is the pulse current amplitude, and *S* is the cross-sectional area of the specimen. Moreover, *I* = *j_e_ S*, where *j_e_* is the peak current density. The initial temperature of the alloy before the electropulsing treatment process was 20 °C. Therefore, the highest temperature of the specimen reached during the electropulsing process at different charging voltages were calculated to be 86 °C, 284 °C, 614 °C, and 1076 °C, respectively, at increasing charging voltage from 2 kV to 8 kV. The temperature increment was low for the specimens treated at the charging voltage of 2 kV and 4 kV, resulting in a faint change of the microstructure. For the 6 kV-treated specimen, the highest temperature reached was 614 °C, which exceeds the onset transition temperature from α to β. The transition phase α″ was first transformed into β phase during the heating process due to the similar crystal structure. Then, during the further holding process or cooling process, the α phase nucleated at the interface of the newly formed β phase and original β phase [26]. Moreover, during the electropulsing process, a large number of electrons moved rapidly along the same direction, forming a kind of electron wind. This electron wind not only can promote the movement of dislocation [15,27], but also forces the diffusion of atoms, as a result of the electromigration effect [28]. The fast heat treatment and the accompanying electron wind caused the transformation process of *α*″ → *β* → *α*. With a further increase in the charging voltage to 8 kV, the specimen was heated to ~1076 °C, which was much higher than the finish transition temperature from α to β. Therefore, a full β phase microstructure was formed during the heating process, which was maintained at room temperature due to the presence of a large amount of β phase-stabilizing element in the alloy. Moreover, a higher degree of superheat due to the superfast heating process also promoted the phase transformation process. One can see that a series of phase transformations occurred under this fast external influence, which inevitably modified the mechanical properties of the alloy, since they occurred during the deformation process [29,30]. 

**Figure 5:**
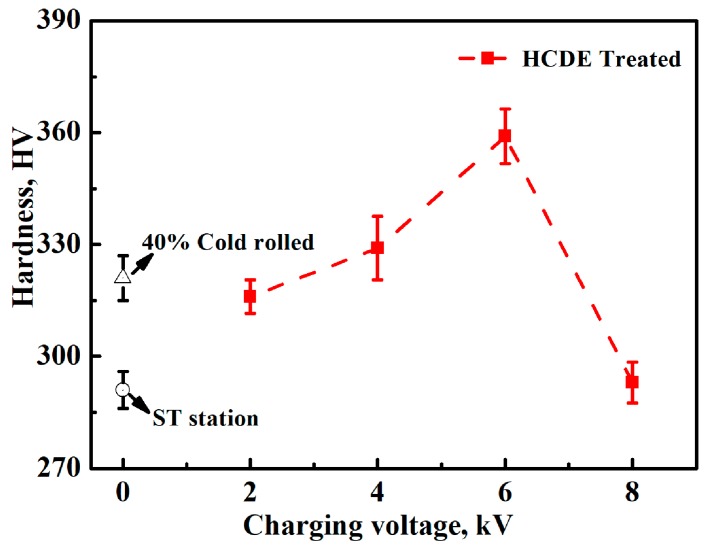
Variation of hardness with charging voltage and comparison with the ST specimen and 40% cold-rolled specimen. HCDE: high-current-density electropulsing.

Figure 5 shows the hardness evolution of the specimen with an increase in the charging voltage. One can see that in comparison to the deformed specimen, the hardness slightly decreased (~5 HV) when the charging voltage was 2 kV. The temperature increment in this specimen was only 66 °C, which was too low to cause an obvious change in microstructure during this extremely short time. The force of the electron wind promoted the dislocation movement, which was likely responsible for the slightly reduced dislocation density within the microstructure and the slightly reduced hardness of the specimen. With a further increase in the charging voltage to 6 kV, the hardness gradually increased to ~359 HV, which was much higher than that of the deformed specimen (~321 HV). Further increasing the charging voltage to 8 kV resulted in a sharp decrease in hardness to ~293 HV, which was nearly the same as that of the ST specimen (291HV). The strength of the transitional phase α″ has been demonstrated to be lower than that of β phase [31], and the strength of the α phase is higher than that of the β phase. Therefore, the specimen treated by 6 kV charging voltage exhibited the highest hardness due to the highest amount of α phase and the disappearance of the α″ phase, while the specimen treated by 8 kV charging voltage exhibited the lowest hardness due to the formation of a full β phase. The notably reduced dislocation density in the 8 kV-treated specimen also contributed significantly to the reduced hardness. It is worth noting that the hardness of the specimen treated by 4 kV charging voltage was higher than that of the cold-rolled specimen, although there was a reduction in the dislocation density. This is probably due to the presence of a small amount of α phase or ω phase formed during the fast cooling process, which could not be detected via XRD because under the detection limit. 

**Figure 6:**
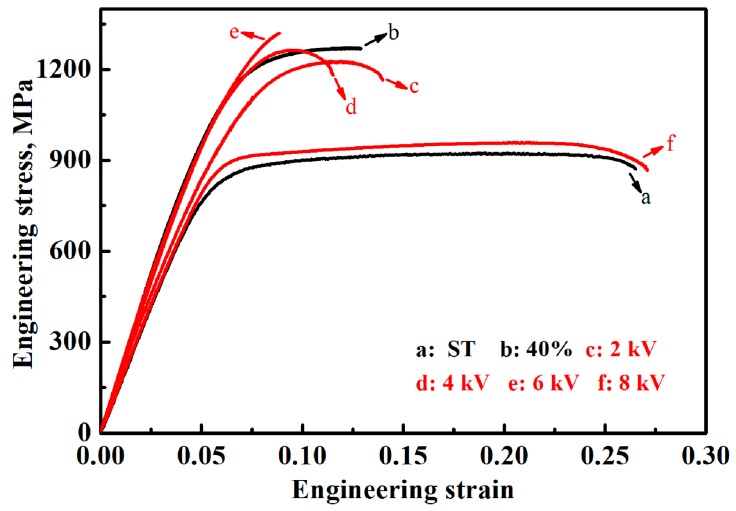
Engineering stress–strain curves of the ST specimen, cold-rolled specimen, and specimens treated by different charging voltages.

Figure 6 shows the engineering stress–strain curves of different specimens, with the detailed mechanical properties being summarized in Table 1. The evolution tendency of the strength of treated specimens is similar to the hardness evolution tendency. With an increase in the charging voltage from 2 kV to 6 kV, the tensile strength increased from 1231 MPa to 1337 MPa, with a gradual decrease in elongation from 7.9% to 1.5%. There was a slight recovery in the specimen treated by 2 kV charging voltage. Therefore, there was a visible reduction in yield strength, a slight reduction in tensile strength, and a slight increase in elongation in comparison to the cold-rolled specimen. A larger amount of α phase formed in the specimen treated by 6 kV charging voltage, directly resulting in a notable increase in strength, as well as a deteriorative ductility due to its high strength. Similar changes were also observed during the conventional heat treatment process, i.e., at a heating temperature of 600 °C followed by furnace cooling or air cooling to room temperature [11] and were attributed to a higher amount of hard α phase. The α phase is stable at room temperature in a zirconium alloy and has an hexagonal close-packed (HCP) structure which usually exhibits a higher strength than the face-centred cubic (FCC) structured β phase [32]. The α phase in a zirconium alloy also possesses good ductility, as in pure Zr and in Zr–Al–Sn alloys [6,33]. Liang et al. also revealed that the α phase deformed first in (α + β) Zr–62Ti–5Al–3V alloy due to the critical resolved shear stresses of dislocation slipping along the basal and prismatic planes in the α-phase, which were 0.15 and 0.12 GPa, respectively, thus lower than the shear stress of 0.24 GPa in the β phase [34]. Therefore, the ductility of the α phase in a zirconium alloy should not be very low. The volume percent of α phase was ~ 23.2% in the 6 kV-treated specimen, but it was ~ 33.9% in the air-cooled specimen obtained by conventional heat treatment at 600 °C [11]. However, the strength of the present specimen was higher than that of the air-cooled one, which indicates a much higher strengthening effect from the α phase in the present 6 kV-treated specimen. Therefore, it is possible that the high amount of β-phase-stabilizing element present in the α phase (e.g., V) increases the strength of the α phase due to the extremely short time left for the diffusion of the element and then it deteriorates the ductility of the α phase and that of the specimen. With a further increase in the charging voltage to 8 kV, the yield strength and tensile strength decreased notably to 817 MPa and 959 MPa, respectively, with a remarkable increase in elongation to 22%. This strength–ductility combination was better than those of the ST specimen and of the specimen treated by the conventional heat treatment process [11] due to the refined grain size, as shown in Figure 2d.

**Figure 7:**
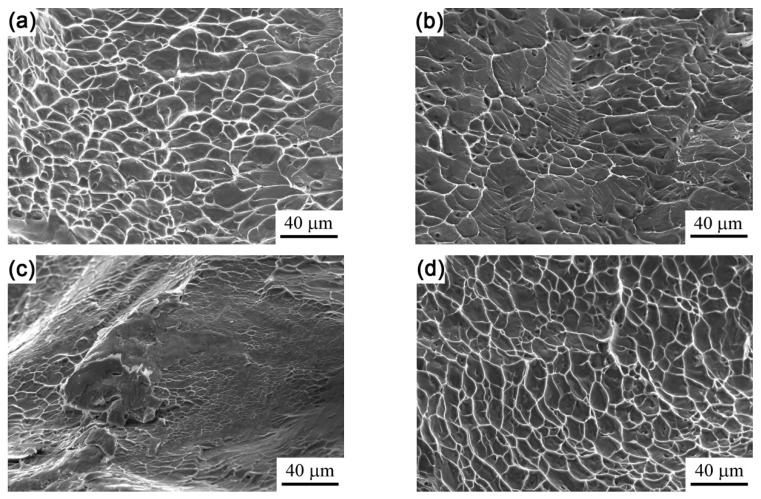
Cross-sectional fractographies of the alloy treated by different charging voltages: 2 kV (**a**), 4 kV (**b**), 6 kV (**c**), and 8 kV (**d**).

Images of the tensile fractographies of these specimens are shown in Figure 7. One can see that ductile dimples were predominant in the fractures of specimens treated by 2 kV, 4 kV, and 8 kV charging voltages, revealing that tension crack propagation occurred in a ductile fracture mode in these specimens. Moreover, the size and depth of the dimples in the fracture of the full-β specimen, i.e., the 8 kV-treated specimen (Figure 7d), were obviously greater than those of other specimens, indicating that the β phase had a better ductility than the α phase. The fracture mode was brittle for the specimen treated by 6 kV charging voltage. The fractography was flat, although there were some shallow dimples. This confirmed the low ductility of this specimen.

## 4. Conclusions

In this paper, the microstructural evolution and mechanical properties of the deformed Zr40Ti5Al4V alloy after the electropulsing treatment process were investigated in detail. The following results were obtained:

(1) When the charging voltage was 2 kV and 4 kV, there was no obvious change in the deformed microstructure., because the highest temperatures reached during the treatment process were only 86 °C and 284 °C, respectively. However, the dislocation density in the microstructure was slightly decreased because the electron wind promoted the dislocation movement, which reduced the strength of the alloy.

(2) When the charging voltage was 6 kV, the highest temperature reached by the specimen was 614 °C. A series of phase transformations occurred during this short time: the α″ phase transformed into the β phase, and the α phase partially formed due to the temperature and electromigration effects. The formation of a stronger α phase notably strengthened the alloy but deteriorated its ductility.

(3) A full β phase could be obtained when the charging voltage was 8 kV. The grain size of the β phase was notably refined to ~26 ± 5 μm. The fast heating rate, short holding time, and fast cooling rate during the electropulsing treatment process were likely responsible for the refined microstructure. This also resulted in a higher strength–ductility combination in comparison to the original solution-treated specimen and the specimen treated by a conventional heat treatment process.

## Figures and Tables

**Table 1:** Mechanical properties of the ST specimen, cold-rolled specimen, and electropulsing-treated specimens.

Specimens	*σ*_0.2_ (MPa)	*σ*_UTS_ (MPa)	*δ* (%)	*φ* (%)
ST	739	922	21.0	22.1
Cold-rolled	1110	1280	7.2	10.7
Charging voltage 2 kV	989	1231	7.9	12.5
Charging voltage 4 kV	1050	1263	5.3	9.6
Charging voltage 6 kV	1164	1337	2.0	1.9
Charging voltage 8 kV	817	959	22.0	35.2
